# Elemental changes in the hippocampal formation following two different formulas of ketogenic diet: an X-ray fluorescence microscopy study

**DOI:** 10.1007/s00775-015-1306-y

**Published:** 2015-11-04

**Authors:** J. Chwiej, A. Patulska, A. Skoczen, K. Janeczko, M. Ciarach, R. Simon, Z. Setkowicz

**Affiliations:** Faculty of Physics and Applied Computer Science, AGH University of Science and Technology, Krakow, Poland; Institute of Zoology, Jagiellonian University, Krakow, Poland; ANKA Synchrotron Radiation Facility, Karlsruhe Institute of Technology, Karlsruhe, Germany

**Keywords:** Ketogenic diet, Hippocampal formation, Topographic and quantitative elemental analysis, X-ray fluorescence microscopy, Synchrotron radiation

## Abstract

The main purpose of the following study was the determination of elemental changes occurring within hippocampal formation as a result of high-fat and carbohydrate-restricted ketogenic diet (KD). To realize it, X-ray fluorescence microscopy was applied for topographic and quantitative analysis of P, S, K, Ca, Fe, Cu, Zn and Se in hippocampal formations taken from rats fed with two different KDs and naive controls. The detailed comparisons were done for sectors 1 and 3 of the Ammon’s, the dentate gyrus and hilus of dentate gyrus. The results of elemental analysis showed that the KDs induced statistically significant changes in the accumulation of P, K, Ca, Zn and Se in particular areas of hippocampal formation and these alterations strongly depended on the composition of the diets. Much greater influence on the hippocampal areal densities of examined elements was found for the KD which was characterized by a lower content of carbohydrates, higher content of fats and increased proportion of unsaturated fatty acids. The levels of P, K and Zn decreased whilst those of Ca and Se increased as a result of the treatment with the KDs.

## Introduction

Recently, diets or metabolic therapies are often applied for the treatment of neurodegenerative diseases such as epilepsy, migraine, brain damage, Alzheimer’s or Parkinson’s diseases, autism, sleep disorders, amyotrophic lateral sclerosis, multiple sclerosis, pain, depression and even cancer [1]. The most famous example of successful diet application against disorders of neurological nature is the use of the ketogenic diet (KD) in patients suffering from intractable epilepsy [2].

Epilepsy, being one of the oldest neurological disorders known to mankind and affecting individuals of all ages, is defined symptomatically by the appearance of spontaneous recurrent seizures [3]. Despite the continued development and release of new antiepileptic drugs, about one-third of people with epilepsy will eventually develop drug refractory seizures [4]. Therefore, there is a great need for better therapeutic strategies. A great body of evidence shows that KD is a broadly effective treatment for drug-resistant epilepsy [5–9]. This high-fat, low-carbohydrate and usually low-protein diet is mostly used to treat pediatric epilepsies but has satisfactory results also in adolescents and adults [10–13].

Catabolism of fats, being the major caloric source in KD, results in the production of ketone bodies which are alternate energy substrates to glucose [14]. The classical formula developed by Rusell and Wilder (1921) is most widely used [15]. It contains long-chain saturated fatty acids as the main component and slight amount of proteins. In this classical formula of the KD, the ratio of fats to proteins plus carbohydrates is 4:1 and is commonly called the ketogenic ratio.

In our previous experiment [16], we investigated effects of the KD on the elemental and biochemical compositions of the hippocampal formation. The diet had its ketogenic ratio set at 9:1. However, it is widely known that various formulas of the KD, having different ketogenic ratios, could be effectively used in clinical antiepileptic therapies [17]. Different health conditions of patients suffering from epilepsy, including children, may be the important reasons to select the diet formulas allowing satisfactory adaptation to the prolonged therapy of those patients who cannot tolerate it well.

Among numerous negative side effects of the long-term KD application, gastro-intestinal problems are frequently observed [18]. Additionally, levels of micronutrients may be reduced, including, for example, a significant deficiency of selenium, causing serious cardiac disorders [19]. This prompts supplementation of the diet with micronutrients to avoid its definitive withdrawal. Therefore, the need arose to develop a less restrictive but equally effective formula of the KD as, for example, the Atkins diet [20].

This is especially important in treating children who tolerate the KD worse than adults [18, 21]. According to clinical practice, diets with higher values of the ketogenic ratio have generally greater capacity to ameliorate epileptic symptoms, but they have stronger adverse effects [17]. Thus, clinical and experimental research focus on optimization of the diet formula leading also to better recognition of components critical to the treatment efficacy. The present study aims to examine and distinguish the effects of two KDs characterized by different ketogenic ratios of approximate values 5:1 (KD1) and 9:1 (KD2).

The model of seizures, however, can significantly hamper detection of the dietary effects. Therefore, to avoid the possible interference, this experimental study was performed on the normal brain which was free of seizures occurring in clinical condition. Such studies have not yet been carried out.

Our previous studies carried out on pilocarpine and electroshock models of seizures showed that elemental anomalies occurring as a result of epileptic activity can be limited only to some areas of the hippocampal formation [22–25]. Therefore, analyzing the diet-induced elemental changes, we should use an analytical tool of high spatial resolving power. Relevant spatial resolution can be achieved with the use of synchrotron X-ray fluorescence technique as reported in previous investigations and reviews [26–29].

## Materials and methods

### Animals and sample preparation

Male Wistar rats came from an animal colony of the Department of Neuroanatomy (Institute of Zoology, Jagiellonian University). All the animal-use procedures applied there were approved by the Bioethical Commission of the Jagiellonian University in accordance with international standards. The animals were maintained under conditions of controlled temperature (20 ± 2 °C) and illumination (12-h light:12-h dark cycle). On the 30th day of postnatal development, the animals were divided into three groups which were afterwards fed either with one of the KDs (KD1 and KD2 groups) or with standard laboratory diet (N group). On the 60th day of postnatal life, the animals were perfused intracardially with physiological saline solution of high analytical quality. The brains were excised, frozen and cut using a cryomicrotome into 12-μm-thick frontal sections. The slices which contained the dorsal part of the hippocampus were mounted on the 4-µm-thick Ultralene^®^ foil. Afterwards, they were freeze-dried and till the measurements stored in the ULT freezer at around −70 °C.

The Ultralene^®^ foil which was used as a sample carrier is free from all, except calcium, the elements under analysis. The content of Ca in the sample was calculated as the difference between the signal from the tissue and bare foil. Moreover, to verify if Ultralene^®^ foil is really free of impurities, the content of metals in blank (foil expanded on the plastic ring) was measured before the analysis of tissue samples. This measurement confirmed the cleanest of the used sample carrier.

The characteristic of the three groups of animals which were examined in the study is presented in the Table 1. Additionally, in the Table 2, the contents of main nutrients, fatty acids and selected elements in the three types of diets are shown.

**Table 1:** The characteristic of examined animal groups

Experimental group	Ketogenic diet 1*	Ketogenic diet 2*	Standard diet	Perfusion**
N (*n* = 7)			+	+
KD1 (*n* = 6)	+			+
KD2 (*n* = 6)		+		+

*n* number of animals in a group

* KDs 1 and 2 were introduced to rats on the 30th day of their postnatal life

** Perfusion with physiological saline solution was done on the 60th day of rat postnatal development

**Table 2:** The content of main nutrients [in (%)], fatty acids [in (g/kg)] and selected elements [in (mg/kg)] in the dry mass of ketogenic and standard diets

Nutrient	KD1	KD2	Standard diet
Lipids	75	79	5
Carbohydrates	5	1	63
Proteins	9	8	25
Others	11	12	7
SFAs*	348	329	–**
MUFAs*	277	330	–
PUFAs*	115	86	–
P	4100	5700	4100
S	160	–	160
K	2200	7900	2200
Ca	7800	7900	7800
Fe	88	138	88
Cu	5.9	11	5.9
Zn	32	51	32
Se	0.41	0.10	0.41

* SFAs, MUFAs, PUFA—saturated, monounsaturated and polyunsaturated fatty acids

** Lack of information concerning the content of selected ingredient

### Experimental method and apparatus

X-ray fluorescence microscopy was used for the qualitative, quantitative and topographic elemental analysis. The measurements were carried out at the FLUO beamline of ANKA [30].

The energy of the beam was tuned to 16 keV using double multilayer monochromator with W-Si multilayers at 2.7 nm period. The beam was focused with the polycapillary into 13 μm spot diameter. The size of the focused beam was measured and optimized by means of knife-edge scans on a silicon chip with 100-nm-thick gold structures. The measured knife edge was fitted to an error function (integral of Gaussian) and the Gaussian parameter sigma was obtained. This parameter was multiplied by 2.35 to get FWHM values.

The flux on the sample was around 10^11^ photons/s. The measurements were carried out in air, at room temperature. Silicon drift detector (Ketek Vitus) in connection with a digital signal processor (XIA, Mercury) was used to obtain the fluorescence radiation spectrum emitted by the sample. The detector was positioned at the angle of 45° in respect to sample and 90° in respect to the exciting beam. The samples were mapped in two dimensions and the time of single fluorescence spectrum acquisition was 8 s. The step size used during raster scanning was equal to 100 µm in both directions and was the compromise between the quality of elemental maps and number of examined cases. It was small enough to obtain the accurate information about the elemental distribution of selected elements in the hippocampal areas of interest and large enough to analyze the whole hippocampal formation in reasonable time what allowed to obtain satisfied statistics of cases. Typically, the number of pixels analyzed per sample was in the range of 800 and 1000 which gave 2–3 h to measure the area of hippocampal formation.

Reference measurements on the following MICROMATTER XRF calibration standards: GaP, CuS_x_, KCl, CaF_2_, Ti, Cr, Fe, Cu, ZnTe, Ge, Se, CsBr, RbI and SrF_2_ were performed for spectrometer calibration and elemental sensitivities calculations.

## Results

**Fig. 1:**
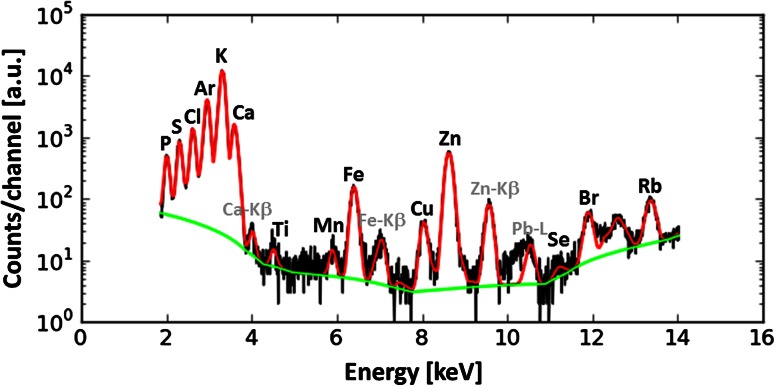
The raw single spectrum recorded in DG area of rat representing N group at the typical measurement conditions (*black line*) together with the fitted spectrum (*red line*) and background (*green line*). The high-energy end of the spectrum including peaks of incoherent and coherent scattering was cut off. The used fitting model took into account K-lines of Ar (present in the air), Ti and Mn as well as Pb-L lines. The occurrence of Ti, Mn and Pb lines in the spectrum probably results from the presence of these elements in the constructing materials used for shields of beamline devices

The analysis of single X-ray fluorescence spectra as well as batch processing of large data sets was carried out using PyMCA software freely available for non-commercial use. One can find the detailed description of the algorithms used in this program in the paper of Sole et al. [31]. In Fig. 1, the exemplary spectrum recorded in the hippocampal formation at the typical measurement condition was presented together with the result of fitting.

The obtained net peak areas of K-α lines of the analyzed elements and elemental sensitivities evaluated based on measurements of calibration standards were used to calculate elemental areal densities for the examined tissue points, which was in detail presented elsewhere [25].

**Fig. 2:**
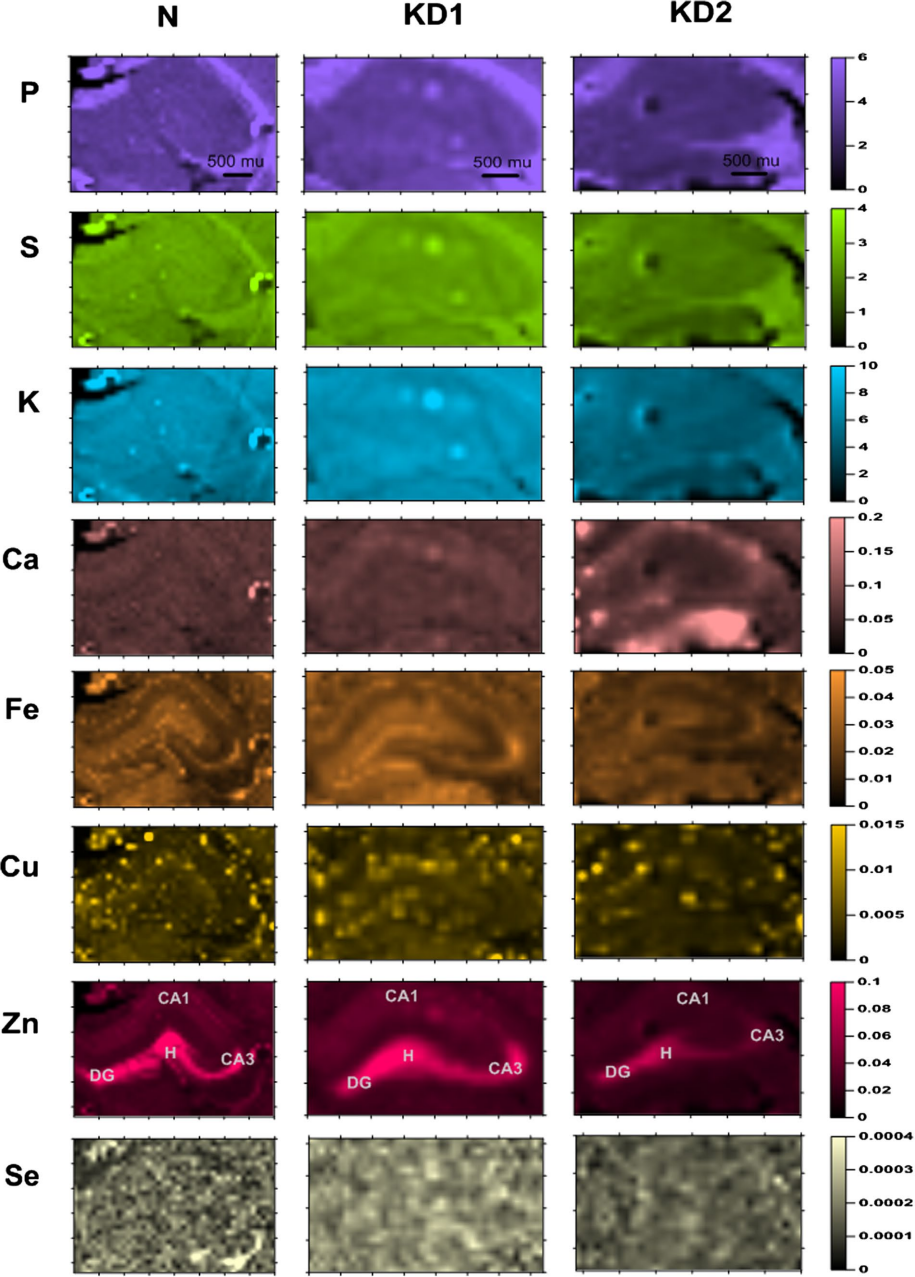
Elemental maps obtained for hippocampal formations from selected animals representing N, KD1 and KD2 groups. The data were interpolated using the Kriging method [32]. Scales display areal densities of the elements in μg/cm^2^

The first step of the study was the topographic analysis of the distributions of: P, S, K, Ca, Fe, Cu, Zn and Se in the analyzed tissue sections. One slice of the dorsal part of the hippocampal formation per each animal (the number of animals used in the study is placed in Table 1) was examined. In Fig. 2, one can see the comparison of the elemental maps obtained for selected rats representing N, KD1 and KD2 groups.

As it can be seen from Fig. 2, most of the elements present quite homogenous distributions within hippocampal formation. The exceptions are Fe and Zn. The increased accumulation of these elements is connected with particular cells or cellular layers. For example, Zn is present at high concentrations in the synaptic vesicles of hippocampal mossy fibers. The preliminary comparisons of the elemental maps obtained for the three examined animal groups showed increased Ca level and decreased Zn level in selected areas of hippocampal formation after the treatment with KD2. As one can see it in Fig. 2, such changes were not noticed for animals fed with KD1.

To confirm statistical significance of the observed changes, the quantitative analysis of selected hippocampal regions followed by appropriate statistical tests was carried out. For all examined samples the mean elemental areal densities were calculated for sectors 1 and 3 of the Ammon’s horn (CA1 and CA3, respectively), the dentate gyrus (DG) and hilus of DG (H). The preliminary identification of the mentioned areas was based on the microscopic view of the unstained slice dedicated to elemental studies. In case of any doubt, additional comparisons with the neighboring slice subjected to hematoxylin–eosin staining were done.

**Fig. 3:**
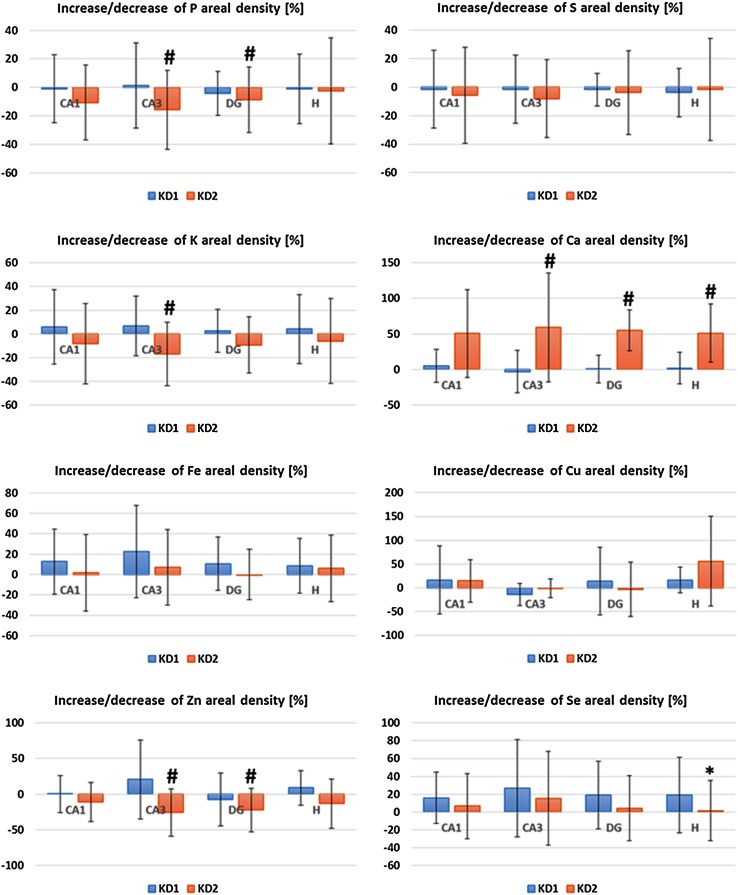
The percentage changes in the elemental composition of CA1, CA3, DG and H hippocampal areas induced by KD1 and KD2. Positive value means higher whilst negative means lower content of element in KD1 or KD2 group comparing to controls on standard laboratory diet. Statistically significant differences between KD1 and N groups were marked with *asterisk* whilst between KD2 and N groups with *hash*. The uncertainties were evaluated according to the law of propagation of uncertainties and the calculations were based on the data presented in the Table 3

One can see the localization of CA1, CA3, DG and H areas within the hippocampal formation in the Zn maps presented in Fig. 2. To compare rats fed with different diets, the median areal densities of elements in these groups were evaluated and presented in Table 3. Statistical significance of differences between medians was tested using non-parametric *U* Mann–Whitney test at the confidence level of 95 %. To better demonstrate KD-induced changes in elemental composition, the percentage differences (PC) in elemental accumulation were calculated according to the formula (1) and are presented in Fig. 3.

1 $$ {\text{PC}}_{\text{KD}}^{e}\, [\% ] = \frac{{{\text{AD}}_{\text{KD}}^{e} - {\text{AD}}_{\text{N}}^{e} }}{{{\text{AD}}_{\text{N}}^{e} }} \times 100 $$

where

**Table 3:**
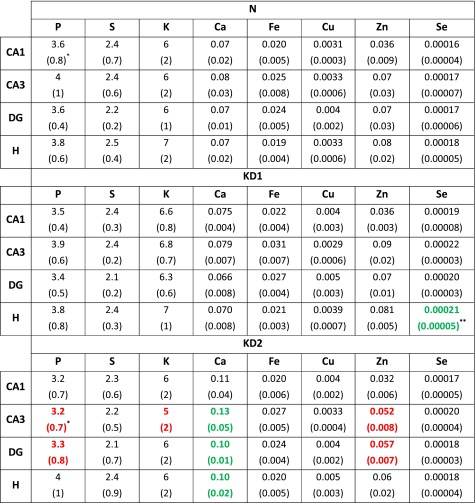
Median areal densities (in μg/cm^2^) of elements recorded for animals on ketogenic (KD1 and KD2) and standard (N) laboratory diets

* The uncertainties of median values were calculated as the interquartile spans and presented in parentheses

** Statistically significant (at the significance level of 5 %) differences found for KD1 and KD2 groups comparing to controls were in bold and, additionally, increases were presented in green whilst decreases in red

PC_KD_^*e*^ is the ketogenic diet (*KD*)-induced percentage change of areal density of element *e*, AD_KD_^*e*^ is the median areal density of element *e* in *KD*-treated animal, AD_N_^*e*^ is the median areal density of element *e* in animal fed with standard laboratory diet.

As one can notice from Table 3 and Fig. 3, KD1 and KD2 introduced different elemental changes in examined areas of hippocampal formation. The rats fed with the KD1 showed increased levels of Se (>15 %) in all examined regions but the only region where this relation was statistically significant was the internal area of DG (19 % increase).

More statistically significant differences were noticed when KD2 and N groups were compared. Higher levels of Ca in CA3, DG and its internal area were found in KD2-fed rats and the percentage increase of Ca areal density was 50 % or more despite the almost equal Ca contents in both diets. The opposite relation was found for Zn. Despite higher Zn content in KD2, the level of this element in KD2 group was significantly diminished in CA3 and DG comparing to animals on standard diet.

Moreover, KD2 group presented lower, in comparison to animals on standard diet, areal densities of P and K. For both elements this relation was statistically significant in CA3 area and for P additionally in DG.

## Discussion and conclusions

In the study, X-ray fluorescence microscopy was used to follow KD-induced changes in elemental composition of the hippocampal formation. The quantitative elemental analysis was done for CA1, CA3, DG and H hippocampal areas. Rats fed with two different ketogenic diets (KD1 and KD2) were compared with normal animals on standard laboratory diet. Commercially available KD2 is characterized by a lower content of carbohydrates, higher content of fats and increased proportion of unsaturated fats in them comparing to KD1. Moreover, KD1 and KD2 differed in the content of some of the analyzed elements, which one can in detail see in Table 2.

Application of KD1 introduced only one change in the elemental composition of hippocampal formation. Higher areal density of Se in the internal area of DG was found in KD1-fed rats comparing to controls. The comparison of KD2 and N groups did not show any differences in Se level what may result from the fact that the content of Se in KD2 was 4 times lower than in the standard laboratory diet.

Selenium being a part of selenoproteins is an essential nutrient of fundamental importance to human biology. Some of the selenoproteins possess antioxidant activities which means that they protect cells against oxidative stress and increase their survival in pathological conditions [33]. Selenocysteine-containing glutathione peroxidases are, together with superoxide dismutases and catalase, the most important antioxidant enzymes [33].

Oxidative stress meaning an imbalance in oxidant and antioxidant homeostasis is strongly implicated in a number of neuronal disorders including Alzheimer’s and Parkinson’s diseases, stroke-related brain damage and seizure disorders [33, 34]. There is also a significant link between Se and epilepsy [33, 35–37]. The plasma level of Se and blood glutathione peroxidase activity were severely reduced in children with intractable seizures [38] and supplementation with Se helped to control seizures [37]. Application of Se reduced tissue damage and normalized EEG [39] in animal model with Fe^2+^-induced epileptic seizures. In the other study, carried out on the pentylenetetrazol model of seizures, it was shown that dietary administration of Se attenuated the breakdown of the blood–brain barrier [40].

Much more statistically significant changes in the elemental composition of hippocampal formation were introduced through KD2. KD2-induced anomalies manifested mostly in CA3 and DG. In both hippocampal areas, lower areal densities of P and Zn and higher levels of Ca were found for KD2 group comparing to controls. Moreover, the decreased level of K in CA3 and increase of Ca in the hilus of DG were noticed in animals fed with KD2.

The most surprising effect of KD2 was the increased level of Ca in all the analyzed hippocampal areas (in CA1 not statistically significant). Although the contents of Ca in the three diets were comparable, hippocampal areal densities of this element increased only in KD2-fed rats for which they were 50–60 % higher comparing to control animals. Such relation was not found for KD1 group which may suggest that an increased Ca level in KD2-fed rats was an effect of the action of the other components of this diet.

The existing literature suggests that polyunsaturated fatty acids (PUFAs) from KD should rather reduce Ca influx and decrease reactive oxygen species production than elevate their levels [5]. This results from the fact that PUFAs induce the activity of mitochondrial uncoupled proteins (UCPs) which leads to a reduction of the proton gradient across the inner mitochondrial membrane [5, 41, 42]. From the other side, according to Hawkes et al., hypercalcemia may be an uncommon complication of the KD [43].

The hippocampal level of Ca increases as a result of seizures [22–24]. Epileptic seizures induce excessive glutamate release which activates postsynaptic NMDA receptors and triggers receptor-mediated Ca^2+^ ions influx [44]. This increase in the concentration of Ca ions promotes a cascade of further events that finally lead to the excitotoxicity and cell death [45].

Therefore, KD2 introduced in the hippocampal areas similar anomalies in Ca accumulation that are observed as a result of seizure activity.

The treatment of animals with the KD2 induced statistically significant decrease of Zn areal density in CA3 and DG areas. Analogous effect was found in our previous study carried out on the pilocarpine model of epilepsy. In the acute phase of pilocarpine-evoked seizures, the level of Zn was reduced in both the mentioned hippocampal areas [22].

It can be assumed that the long-term influence of KD on the nervous tissue produced changes similar to those occurring after the onset of seizures. Consequently, these changes could modify so much the properties of the tissue that it was no longer able to develop a complete seizure response after administration of the chemoconvulsant. These changes appear to resemble preconditioning phenomena described in numerous reports, including that by Kosonowska et al. [46]. The present paper does not identify the mechanisms underlying the observed phenomenon, but may suggest that their action may not necessarily be of neuroprotective nature.

Zinc is an essential nutrient which can be supplied to the brain through the blood–brain and blood–cerebrospinal fluid barriers [47]. Although there is many links between Zn and epilepsy, the role of this element in the etiology and manifestations of seizures has still been very enigmatic [47, 48]. The intracerebroventricular administration of Zn ions causes epileptic seizures in rats and their increased level was found in the inborn audiogenic mouse [49, 50]. On the other hand, subcutaneous injections of this element reduce the frequency of noise-induced clonic and tonic seizures and deaths in mice, but had no significant effect on the occurrence and severity of kainic acid-induced seizures in rats [51]. Fukahori et al. showed, moreover, that dietary Zn loading diminished, whilst Zn deficiency increased seizure susceptibility in epilepsy mice [52]. Therefore, as it can be easily seen, zinc in seizures may have both pro- and anticonvulsant effects. Moreover, this dual action appears to be dose dependent [48].

The higher level of Ca and lower areal density of Zn which were observed in KD2-fed animals point that KD may induce in the hippocampal formation similar conditions as epileptic seizures do. Therefore, anticonvulsant activity of KD does not have to be associated with its neuroprotective effect on the brain. Another reason is that the KD was introduced in animals which were too young and, therefore, its long-term application disturbed their brain development. Also, it appears possible that some other mechanisms are involved in the action of KD and the diet targets other cellular elements of the nervous tissue including astrocytes. For detailed exploration of the problem, further studies are necessary.

The results presented in the study did not show any KD-induced changes in S, Fe and Cu hippocampal accumulation. Animals fed with KD2 presented lower, in comparison to animals on standard diet, areal densities of P and K. For both elements this relation was statistically significant in CA3 area and for P additionally in DG. The change of K level may be connected with the action of PUFAs and/or ketone bodies. Recently, it has been suggested that PUFAs, in conjunction with ketone bodies, may activate a lipid-sensitive class of K_2_P potassium channels and enhance the activity of the Na+/K+-ATPase (sodium pump) [6, 53]. In this context, it is necessary to mention that both increased accumulation of ketone bodies and increased unsaturation level of lipids in the hippocampal formations were previously found for rats fed with KD1 and KD2, which was in detail described elsewhere [16, 54].

Assuming, the results presented in this paper showed that KD may induce significant changes in accumulation of selected elements. The changes depend on the composition of high-fat diets and seem to be much stronger in case of the diet with higher ketogenic ratio.
